# Int Braz J Urol Annual Report - 2017

**DOI:** 10.1590/S1677-5538.IBJU.2018.0001

**Published:** 2018

**Affiliations:** Professor Titular Disciplina de Urologia da Faculdade de Medicina do ABC, Santo André, SP, Brasil

Traditionally, every year, the International Brazilian Journal of Urology evaluates its reviewers and selects the most efficient along the previous year, Evaluation is based on the number of performed reviews, time for conclusion and quality of work (1).

In 2017, the five most efficient reviewers were:

Fabio Vicentini (Hospital das Clínicas da Faculdade de Medicina da USP, SP, Brasil), Eduardo Kaiser Ururahy Nunes Fonseca (Hospital Israelita Albert Einstein SP, Brasil), Victor Srougi (Faculdade de Medicina de São Paulo, SP, Brasil), Elcio Silva (Clínica Dr. Élcio Dias Silva, Campinas, SP, Brasil) and Kemal Sarica (Kartal Dr. Lufi Kirdar Training and Research Hospital Istanbul, Turkey).

We would like to thank them and all others reviewers, that are the reason for the existence of a peer-review scientific journal.

Int Braz J Urol is an open-access magazine, integrally supported by the Brazilian Urological Society, that does not charge for the submission of papers. Also, it is included in the Urology Green List (2), that reunites all reliable and safe urological journals on which the urological community can publish their researches.

In 2017, our Journal received 603 papers, and 28% were accepted. Medium time for first decision correspondence was 44 days, and more 42 days for publishing as Ahead of Print. Papers were sent from 42 countries, from 635 different institutions and 2254 authors; we used 1135 reviewers. The 5-year impact factor of IBJU in 2017 was 1.097.
